# Relationships Between Pilots’ Startle and Surprise Responses and Information-Processing Performance During Simulated In-Flight Events

**DOI:** 10.1177/00187208261434426

**Published:** 2026-03-25

**Authors:** Jiayu Chen, Annemarie Landman, Alexis Derumigny, Olaf Stroosma, M. M. (René) van Paassen, Max Mulder

**Affiliations:** 1 2860 Delft University of Technology, The Netherlands; 2 China Automotive Engineering Research Institute Co., Ltd, China; 3 312992 TNO, The Netherlands

**Keywords:** aviation, cognition, stress, working memory, incapacitation

## Abstract

**Objective:**

We aim to investigate how pilots’ startle and surprise responses affect information-processing performance during simulated in-flight events.

**Background:**

Startle and surprise are distinct constructs, each with their own potential effects on pilot’s performance during unexpected in-flight events. Prior research suggests that startle may impair performance through stress-induced cognitive interference, whereas surprise may do so via cognitive demands associated with sensemaking. Thus, we hypothesized that both startle and surprise would negatively affect information-processing performance on a secondary auditory cognitive task.

**Method:**

Using a motion-based hexapod simulator and a twin-propeller aircraft model, 26 pilots each performed eight single-pilot flight scenarios, which were designed to elicit varying levels of startle and surprise responses. Linear mixed-effects models were employed to analyse the relationships between self-report startle and surprise with secondary task performance, while controlling for individual differences and differences between the scenarios.

**Results:**

The results revealed that higher startle was significantly associated with reduced information-processing speed. For surprise, no significant association was found.

**Conclusion:**

The findings suggest that, within the context of the tested scenarios, startle appeared to impose a more pronounced disruptive effect on pilots’ information-processing performance than surprise.

**Application:**

The study underscores the need for tailored interventions to enhance pilots’ resilience to startle and calls for further research on ecologically valid methods to induce surprise for research and training purposes.

## Introduction

During non-normal in-flight events, high workload and pressure may pose severe challenges to pilot cognitive functioning. Cognitive performance impairments such as attentional tunnelling, break-down of teamwork, hasty decision making, or automation mode confusion have been described (De Boer & Dekker, 2017; Parasuraman et al., 2000; Sarter et al., 2017; Wickens, 2008; Young & Stanton, 2002). These challenges may be exacerbated when pilots experience startle and surprise (Casner et al., 2013). Recognizing this impact, the European Union Aviation Safety Agency and Federal Aviation Administration have integrated startle and surprise into the regulatory framework for Upset Prevention and Recovery Training (UPRT) to mitigate risks (European Aviation Safety Agency, 2015; Federal Aviation Administration, 2015).

Startle refers to a coinciding emotional and physiological response to abrupt, intense stimuli perceived as potentially threatening. It is characterized by involuntary physiological startle reflexes and generalized stress responses (Dreissen et al., 2012; Holand et al., 1999; Koch, 1999; Martin et al., 2015). The startle reflex, typically within 100 milliseconds after a stimulus, includes eye-blink, head movement, facial grimacing, shoulder elevation, arm abduction, elbow bending, forearm pronation, finger flexion, and abdominal contraction (Ladd et al., 2000; Leuchs et al., 2019). If the threat persists, it triggers a generalized stress response involving activation of the autonomic nervous system and the release of cortisol (Martin et al., 2015). This leads to rapid breathing, increased heart rate, systolic blood pressure, pupil dilation, and amplified sensory arousal (Dreissen et al., 2012; Holand et al., 1999; Jansen et al., 1995; Papadimitriou & Priftis, 2009).

Research has shown that startle and resulting stress can temporarily disrupt cognitive processes, impair sustained attention, and reduce cognitive efficiency (Sehlström et al., 2022; Valls-Sole et al., 2008). Neurobiological models indicate that startle activates survival circuits, prioritizing threat detection at the expense of cognitive processing, thereby interfering with executive function (LeDoux, 2012). This disruption could limit working memory and attentional control by diverting cognitive resources toward stimuli-driven processing, thereby compromising goal-directed behaviour (Eysenck et al., 2007). This overload may narrow attention, reduce situation awareness, impair decision making and information-processing capacity (Casner et al., 2013; Dehais et al., 2013). The severity and duration of these effects vary depending on individual differences (Bradford et al., 2014), contexts, and stimuli (Lang et al., 1990). The disruptions are particularly concerning in high-risk operational domains such as aviation, where precise and timely decision making is critical (Landman et al., 2017; Rivera et al., 2014). Empirical evidence demonstrates that unexpected in-flight events can elicit elevated physiological arousal (e.g. increased heart rate and pupil dilation), impair processing of critical information, and degrade overall performance (Kinney & O’Hare, 2020; Martin et al., 2016). While several studies have documented transient cognitive impairments following startle (Deniel et al., 2024), others have reported minimal effects or even performance enhancements under high cognitive load conditions (Schwartz et al., 2025). These mixed findings underscore the importance to investigate the effect of startle within highly ecologically valid contexts.

Surprise is a cognitive and affective response triggered by unexpected, schema-discrepant events that are (momentarily) difficult to explain (Horstmann, 2006; Meyer et al., 1997). Surprise interrupts ongoing automatic cognitive processes, directing attention towards analysing unexpected events, and updating one’s understanding of situations (Reisenzein et al., 2019). This interruptive effect has been quantified with the latency of verbal and motor response tasks in labs (Wessel & Aron, 2017). The sensemaking and “reframing” processes require effortful, goal-directed cognitive processing (Klein et al., 2007; Landman et al., 2017). Goal-directed processes that are required for sensemaking are susceptible to disruption under high stress, which could hinder the acquisition of relevant information and the execution of appropriate actions (Eysenck et al., 2007; Landman et al., 2017).

Thus, although startle and surprise are theoretically and psychometrically distinct constructs (Chen et al., 2025a, 2025b), both startle and surprise may impair pilots’ information-processing performance by disrupting ongoing cognitive processes. Startle could induce additional mental workload by diverting attention to the startling stimuli, or due to rapid engagement of survival circuits that prioritize a quick response to threat over executive functioning (LeDoux, 2012). Surprise could impair information-processing by imposing additional demands on working memory to make sense of the surprising events.

Therefore, we aim to investigate the relationships between subjective startle and surprise severity on pilots’ information-processing performance. Building upon research performed in lab settings (Duchevet et al., 2025), the approach will be to use a more ecologically valid context consisting of single-pilot basic flying tasks performed in a motion-based simulator. Using a dual-task paradigm (Wickens, 2002), information-processing performance is quantified using a secondary auditory cognitive task, that was performed concurrently with in-flight events. An auditory task was chosen to not interfere with visual information processing.

We hypothesize that both startle and surprise would impair information-processing performance, reducing secondary task performance. To test the hypotheses, pilots perform eight flight scenarios designed to elicit a wide range of startle and surprise responses. The objective is to test whether higher levels of startle or surprise are associated with poorer secondary task performance, while controlling for scenario differences and general individual differences in performance. To measure startle and surprise, the developed and validated multi-item Startle Inventory and Surprise Inventory are employed, respectively (Chen et al., 2025a, 2025b). Insights into the effects of startle and surprise on information processing in a context that is relatively high in ecological validity may inform the development of training programs aimed at enhancing cognitive resilience during in-flight emergencies.

## Method

### Participants

A total of 26 professional pilots (25 males, 1 female) participated in the study. Among them, 18 held a full Airline Transport Pilot License (ATPL), and 8 held a frozen ATPL, indicating they had completed all theoretical exams under the EASA licensing framework but had not yet accrued the required 1,500 flight hours for issuance of the full license. In terms of current professional roles, the participants included 14 captains, 8 first officers, 3 second officers, and 1 pilot employed in a non-airline aviation position.

**Figure 1.**
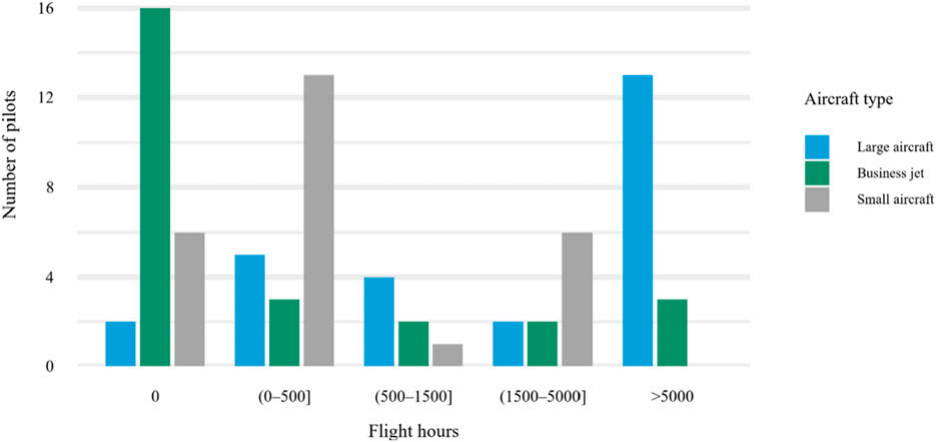
Distribution of pilots’ flight hours across aircraft types (N = 26). Each pilot may have experience in multiple aircraft types. No pilot reported *> *5000 flight hours on small aircraft

The characteristics of participants are summarized in Table 1. Figure 1 illustrates the distribution of flight hours across different aircraft types (N = 26). All participants provided informed consents. This research complied with the American Psychological Association Code of Ethics and the Research Ethics Committee of the Delft University of Technology approved the research design (No. 4056).

**Table 1.** Characteristics of the participants

	Mean	*SD*	Min	Max
Age (yrs)	43.8	13.0	23.0	67.0
Employed time (yrs)	17.8	13.2	0.5	44.0
Flight hours	8633.9	7082.1	280.0	25500.0
Large aircraft	6566.1	6607.8	0.0	22000.0
Business jet	1257.7	2803.7	0.0	10000.0
Small aircraft	810.1	1258.4	0.0	5000.0

### Apparatus

**Figure 2.**
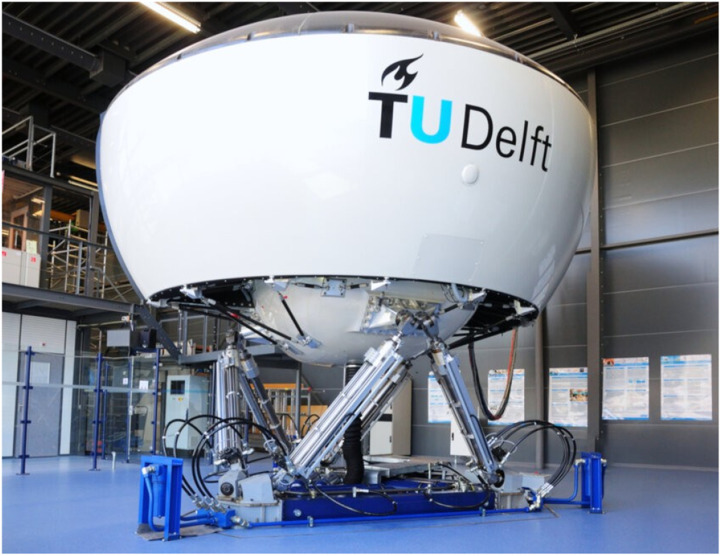
The SIMONA research simulator

The experiment was performed using the SIMONA Research Simulator at the Delft University of Technology (Figure 2). This is a full-motion simulator equipped with a hydraulic hexapod platform providing six degrees of freedom. The simulator has a collimated 180 degrees horizontal by 40 degrees vertical field of view for outside vision rendered with FlightGear. A 5.1 surround sound system was installed for realistic 3D sound effects of potential startling or surprising events, alarms, flaps, retractable gear, aerodynamic noise, ground rumble, and engines. During the experiment, participants wore single-ear intercom headsets.

**Figure 3.**
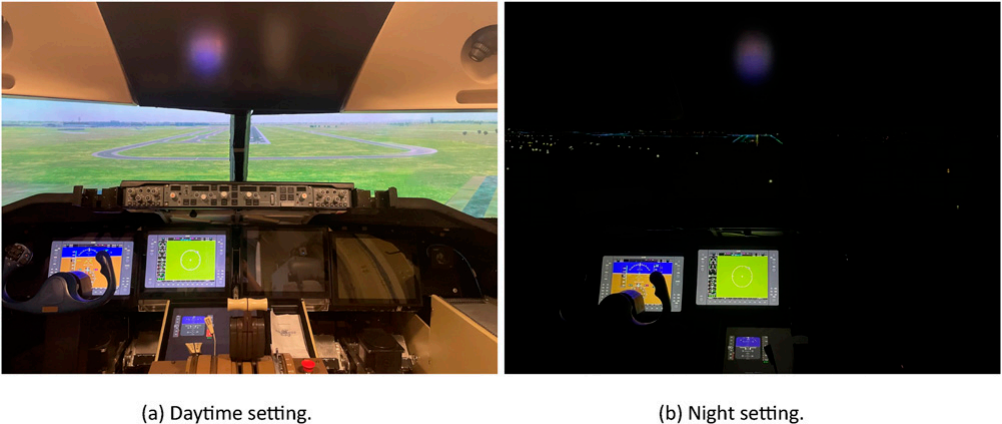
The experimental setup (simulated flight deck)

The experiment employed an aerodynamic model of the Piper PA-34 Seneca III, a light twin-propeller aircraft. The flight deck (see Figure 3 for daytime and night settings) featured flight controls, including a control column with pitch trim, rudder pedals with force feedback, throttle, gear, and flaps with three settings: 0° (UP), 25°, and 40° (LAND). The avionics consisted of a primary flight display (PFD) similar to a G1000 PFD, a backup primary flight display, and a multifunction display for engine, configuration, and navigation data. Information on airspeed, altitude, attitude, engine parameters, flaps position, and gear status was available via the avionics displays.

### General Procedure

**Figure 4.**
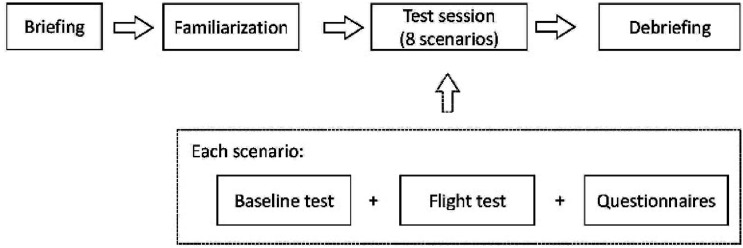
The experimental procedure

An overview of the experimental procedure is illustrated in Figure 4. Pilots performed tasks on a single day and as single-pilot crew. The total duration of the experiment per participant was approximately 2 hours, including the briefing, familiarization, test session (comprising eight test scenarios), and debriefing.

**Figure 5.**
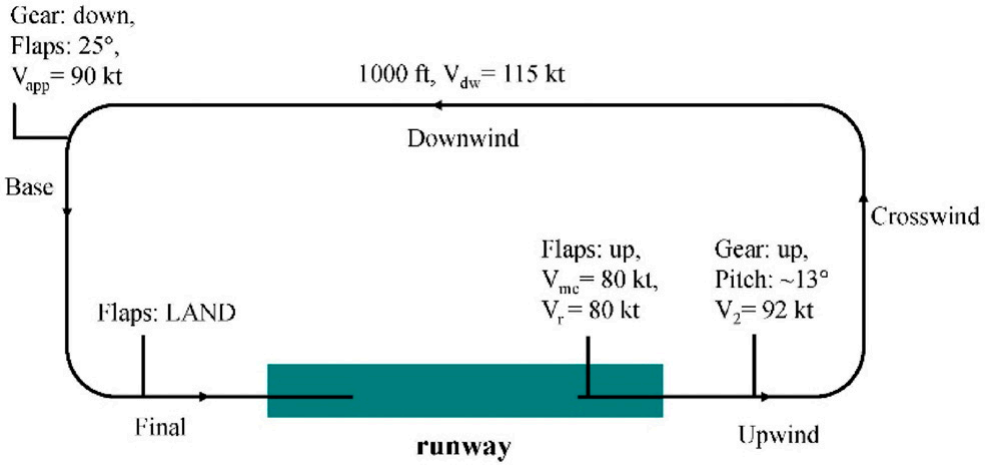
The standard traffic circuit with target settings

All pilots were briefed about the aircraft model, simulator features, experimental tasks, and definitions of startle and surprise. Each familiarization and test scenario required the pilot to fly (part of) a left-handed traffic circuit (Figure 5) for runway 18C, Schiphol Airport (EHAM). The circuit would need to be flown at 1,000 ft with a speed of 115 kt. A flaps setting of 0 (UP) was required for take-off, 25 during base leg, and 40 (LAND) in final. The circuit required rotate speed of 80 kt, minimum control speed of 80 kt, best rate of climb speed of 92 kt, and landing approach speed of 90 kt. These configurations were also available on kneepad in the simulator.

During familiarization, pilots also practiced the secondary auditory task (see the Secondary Auditory Task section) once on the runway, and once while performing the circuit. At the end of the familiarization, all pilots confirmed that they could handle the aircraft model, none required help in determining the turn points of the circuit, and none had difficulty with performing the secondary auditory task.

Pilots then proceeded with the test session, which consisted of eight test scenarios (see the Startle and Surprise Events section), presented in a semi-counterbalanced order defined by a Latin square (Hinkelmann & Kempthorne, 2007). Test scenarios began either from the take-off position on runway 18C, or in-flight position at 800 ft ahead of runway 18C, with an airspeed of 99 kt. In all cases, participants were required to complete the circuit and land safely on the same runway.

Before each scenario, participants received a briefing on wind strength, direction, and weather code through a Meteorological Aerodrome Report (METAR). Before and during each scenario, pilots performed blocks of the auditory task (see the Secondary Auditory Task section). Immediately following each scenario, pilots completed a questionnaire that included the Startle and Surprise Inventories (see the Dependent Measures section). After completing all scenarios, pilots were informed about all simulated events in a debriefing.

### Startle and Surprise Events

Test scenarios were designed to induce relatively high surprise or high startle, both, or neither, based on the characteristics of the preset events. This was done to maximize variation between scenarios in the pilots’ responses, which is necessary for investigating relationships between the responses and information-processing performance. Events that were rare, unfamiliar, or difficult to immediately explain were used to induce relatively high surprise (Izard et al., 1993; Klein et al., 2007; Landman et al., 2017; Meyer et al., 1997). Events that were sudden, loud, or immediately threatening were used to induce relatively high startle (Blumenthal, 1988; Bradley et al., 2005; Koch, 1999; Martin et al., 2015). More “high surprise” than “high startle” scenarios were included because we expected difficulty with surprising a sufficient proportion of pilots to induce sufficient variation for analysis. The characteristics of the events and secondary auditory tasks are listed in Table 2.

**Table 2.** The characteristics of event and secondary auditory task in each test scenario

ID	Event description	Event inserted^a^	Lead time^b^	Event timing	Non-target (distraction) block timing
ENF	The right engine failed shortly after take-off.	Right engine failure during take-off	10 s	5 s after reaching 900 ft	Climbing through 100 ft
FLAP	When selecting Flaps 25 in the base leg, the left flap remained UP.	The aircraft response when you selected Flap 25	5 s	At Flaps 25 selection	Climbing through 800 ft
CARGO	Cargo moved backward during take-off with a loud sound and pitch-up motion.	The pitch-up motion (and noise) after rotation	15 s	10 s after reaching 200 ft	-
LTS	While flying at night, lightning struck the aircraft with a bright flash and loud thunder sound.	The lightning strike	5 s	5 s after descending to 500 ft	Descending through 800 ft
PFDF	The PFD turned black.	The malfunction of the PFD	5 s	Descending to 600 ft	-
STALL	A bird strike triggered a false stall alarm with stick shaker.	The stall alarm	5 s	20 s after reaching 800 ft	Climbing through 150 ft
NTO	Normal take-off without preset malfunction.	The level-off manoeuvre	-	-	-
NLO	Normal landing without preset malfunction.	The effect of crosswind	-	-	-

*Note.* PFD = primary flight display.

^a^The specific content that replaced the placeholder ‘[the stimulus]’ in the Startle and Surprise Inventories.

^b^Lead time as in Figure 6b.

The following three scenarios required pilots to manually respond to controllability issues. In the engine failure scenario (ENF), the right engine failed during climb after reaching 900 ft, causing a roll and yaw moment that could be counteracted using the column and pedals. The pilots’ unfamiliarity with the specific aircraft response to the engine failure was expected to induce surprise. Since there was no immediate threat nor intense stimuli, a limited startle response was expected. In the flaps asymmetry scenario (FLAP), the left flap remained in the UP position when pilots selected Flaps 25 during the turn to base leg. This caused an unexpected roll and yaw moment, which need to be counteracted using the control column. The pilots’ unfamiliarity with the specific aircraft response to the flap failure was expected to induce surprise. Since there was no immediate threat nor intense stimuli, a limited startle response was expected. In the cargo shift scenario (CARGO), a simulated piece of heavy cargo broke loose and shifted towards the tail after take-off, with a loud scraping and collision noise coming from the back of the aircraft. This event temporarily moved the aircraft’s centre of gravity backward, resulting in a violent pitch-up motion that pilots had to correct using the control column. The novelty of the event and difficulty in explaining it were expected to induce high levels of surprise, while the sudden (upset) motion of the aircraft and the accompanying loud scraping noise were expected to induce startle response.

The following scenarios required no intervention from pilots and featured no imposed changes to simulator or aircraft motions. The lightning strike scenario (LTS) started at the in-flight position in the night condition, with weather report indicating the presence of thunderstorms. The scenario was designed to be highly startling due to the sudden bright flash and loud thunder sound, but not (limited) surprising due to the stated weather conditions. Between 400 and 500 ft, a lightning strike was simulated (Chen et al., 2025b), accompanied by a thunder sound played over a surround sound system, and a strobe light flash presented via the simulator’s projection system.

The second no-intervention scenario was the primary flight display failure scenario (PFDF). Due to its unfamiliar nature, this scenario was intended to elicit a high level of surprise. However, in the absence of intense stimuli or immediate threat, a limited startle response was expected. It started at in-flight position. The PFD turned to black at 600 ft. Pilots could use the outside view and the backup display to continue landing. The third no-intervention scenario was the false stall warning scenario (STALL). After take-off, a bird struck the angle of attack at 800 ft, triggering a continuous false stall alarm. Due to the lack of context for a stall event, it was expected to be surprising, and due to the sudden loud auditory stall alarm and stick shaker it was expected to be startling.

Finally, two more scenarios, normal take-off (NTO) and normal landing (NLO), were included to present events that were expected to induce low levels of startle and surprise. NTO started at the take-off position and NLO started at the in-flight position, with pilots performing a landing under a 5 kt crosswind from the east.

### Secondary Auditory Task

The pilots were informed that the auditory task was designed to assess their capacity to process auditory information. In line with standard procedures, they were instructed to always prioritize aircraft control over the auditory task.

**Figure 6.**
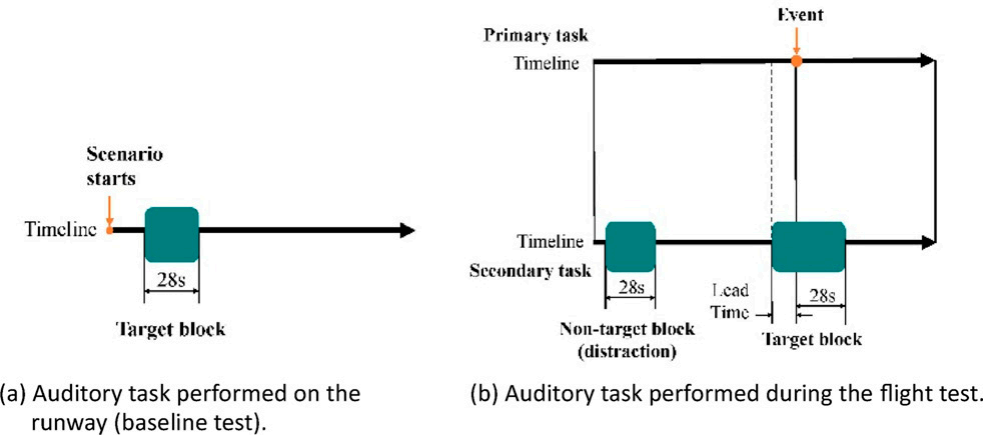
Auditory tasks in the familiarization and test session

A “block” consisted of ten randomly generated numbers, ranging from 0 to 9, pronounced in the ICAO Phonetic Alphabet, where presented over the pilots’ headset with 2.5 second intervals resulting in a total block duration of 28 seconds. Each block was preceded by an auditory warning: “The auditory task is coming”. The target block, where performance was collected, started at 5, 10, or 15 seconds before the preset event with 2, 4, or 6 additional numbers, respectively, and always continued for 28 seconds (10 numbers) after event onset (see Table 2 and Figure 6).

Non-target (distraction) blocks were also included in the ENF, FLAP, STALL, and LTS. These were presented at different moments than the startle/surprise events (i.e. non-target (distraction) block timing in Table 2) and always lasted 28 seconds. Their only purpose was to reduce participants’ expectation of startle/surprise events, which were consistently paired with target blocks.

Participants were instructed to single-click the autopilot disconnect button with their thumb if the number was odd, and to double-click it if the number was even. For double-clicks, the interval between clicks had to be less than 500 milliseconds. Otherwise, the response would be recorded as one single-click and one invalid click.

Both aircraft control and auditory task were implemented in the Delft University Environment for Communication and Activation (DUECA). This ensured synchronisation between flight phase, auditory stimulus presentation, and responses acquisition.

### Dependent Measures

#### Auditory Task Reaction Time

The main measure of the information-processing performance was the auditory task reaction time. The autopilot disconnect button was sampled at a frequency of 100 Hz, after which reaction times were calculated with a resolution of 0.01 seconds. The mean reaction time of the correct responses was obtained for the target block (after the event) and for the baseline test for each scenario. To clean the data, responses that were either extremely fast (i.e. quicker than the average response time in baseline) or missing, were excluded as invalid. The mean reaction time during the flight test was then corrected by subtracting the mean baseline reaction time (obtained on the runway), resulting in the Delta Reaction Time (Δ*RT*).

#### Auditory Task Accuracy

As additional measure of information-processing performance, accuracy within the target block (after the event) was assessed. It was defined as the ratio of correct responses to the total number of presented numbers in the target block (i.e. 10). To account for individual baseline performance, flight test accuracy was baseline-corrected by subtracting the baseline accuracy (obtained on the runway), yielding the Delta Accuracy (Δ*AC*).

#### Measures of Startle and Surprise

Following each test scenario, participants completed the Startle Inventory (Startle-I) and Surprise Inventory (Surprise-I) regarding each in-flight event (Table 2). The Startle-I and Surprise-I are validated self-report measures designed to assess startle and surprise responses to specific stimuli (Chen et al., 2025a, 2025b). The Startle-I consists of six statements: “It startled me.”, “It made me physically flinch.”, “It caused my heart to suddenly beat harder or faster.”, “It immediately made me feel scared or angry.”, “It shocked me.”, and “It immediately caused stress or frustration to me.”, to which the participant rates agreement on 1–5 Likert scales (1 = Strongly disagree, 2 = Disagree, 3 = Neutral, 4 = Agree, 5 = Strongly agree). These statements aim to capture operationally meaningful levels of startle, rather than transient startle reflexes. The Surprise-I consists of five statements: “It surprised me.”, “It was consistent with my expectations.” (reverse-coded), “I predicted it beforehand.” (reverse-coded), “I did not see it coming.”, and “It was unexpected.”, which are scored in the same way as the Startle-I. The total score of each inventory is the average of the items’ scores, ranging from 1 to 5.

McDonald’s *ω* (McDonald, 2013) for the current sample ranged from *ω* = 0.88 to *ω* = 0.96 for the Startle-I, and *ω* = 0.77 to *ω* = 0.96 for the Surprise-I across scenarios, indicating acceptable to excellent internal consistency.

### Statistical Analyses

To account for the repeated-measures design and non-independence of observations across both participants and scenarios, a modified ‘between and within formulation’ (Hox et al., 2010) was applied. For each dependent variable, a separate linear mixed-effects model was fitted, to control for the participant-level and scenario-level and variability.

For testing our hypotheses on the relationships between perceived startle and surprise with Δ*RT* or Δ*AC* across different scenarios, heteroscedastic linear mixed-effects models were applied. The fixed effects were the ratings of startle (*Startle-I*), the ratings of surprise (*Surprise-I*), and scenario (*Scenario*) modelled as a categorical variable with eight levels. This means that potential scenario effects on Δ*RT* or Δ*AC* were controlled for. Additionally, if the sequence of scenarios (*Sequence*) was found to have a significant effect on Δ*RT* or Δ*AC*, the sequence of the scenarios was also included in the linear mixed-effects model, to control for this as well. To account for general individual differences in Δ*RT* or Δ*AC*, participant number (*ID*) was included as a random effect.

Linear mixed-effects models were fitted using the lme function from the nlme package in R. To account for heteroscedasticity in the residuals, the varIdent function was used to accommodate variance differences across scenarios. Effect size was measured as the value of an (estimated) coefficient divided by the total variance. This total variance was estimated as the sum of the between-participants variance and the (average) within participants variance (where the average was taken with respect to all possible scenarios).

Furthermore, the Intraclass correlation coefficient (ICC; Raudenbush & Bryk, 2002) was calculated to assess the proportion of the total variance attributable to differences between participants. The ICCs were derived from random effect results, with a higher ICC indicating the notable between-participant variability, supporting the application of linear mixed-effects models.

As additional exploratory analyses, three correlation matrices were computed: (1) between-participant correlations, using participant-level random effects; (2) between scenario correlations, based on estimated marginal means within scenarios; and (3) residual correlations, derived from model residuals. We added age and flight hours to the matrices, as it would be interesting to check if, for instance, more experienced pilots reported lower surprise overall in the experiment. The between-scenario correlations were obtained to check if, for instance, more startling scenarios also affected secondary task performance more. This is highly exploratory as the number of pairs is very low (*n* = 8) and significant correlations are likely to be confounded by other scenario differences.

Finally, to visualize the temporal pattern of pilot performance during the different scenarios, the mean and standard deviation of Δ*RT* were calculated across the sequence of ten numbers, as well as the proportion of valid responses for each number in the sequence.

## Results

### Relationships Between Startle and Surprise with ΔRT

No significant effect of the scenario sequence (*Sequence*) on Δ*RT* was found; hence, it was not included in the model. The linear mixed-effects model of Δ*RT* was fitted as follows:

ΔRT=1+Startle−I+Surprise−I+Scenario+(1|ID).

The results of the linear mixed-effects model of Δ*RT* are presented in Table 3. Higher Startle-I scores were significantly associated with an increase in Δ*RT*, *β* = 0.049, *SE* = 0.017, *t*(171) = 2.817, *p* = 0.005. This indicated that for every point scored higher on the Startle-I, Δ*RT* increased by 49 ms, after controlling for the effect of *Surprise-I*, *Scenario,* and *ID*. In contrast, no significant effect of the *Surprise-I* was found on Δ*RT*.

**Table 3.** Summary of the linear mixed-effects model of the Delta Reaction Time

Effect	Estimate	*SE*	*t*	*p*	Variance factor^a^	Effect size
Fixed effects						
(Intercept)	0.065	0.036	1.8243	0.070	-	0.486
Startle-I (1–5)	0.049	0.017	2.817	0.005**	-	0.363
Surprise-I (1–5)	0.002	0.012	0.139	0.890	-	0.012
Scenario_ENF_	0.257	0.053	4.849	<0.001**	2.128	1.906
Scenario_FLAP_	0.131	0.060	2.181	0.031*	2.756	0.974
Scenario_CARGO_	0.137	0.058	2.379	0.019*	2.547	1.017
Scenario_LTS_	−0.107	0.046	−2.315	0.022*	1.162	−0.796
Scenario_PFDF_	−0.008	0.039	−0.202	0.841	1.341	−0.058
Scenario_STALL_	0.076	0.055	1.388	0.167	2.155	0.567
Scenario_NLO_	−0.045	0.023	−1.981	0.049*	0.895	−0.333
Random effects (*SD*)						
Between-participant	0.073	-	-	-	-	-
Within-participant (NTO)	0.086	-	-	-	-	-

*Note.* Number of observations = 206; Coefficients for each Scenario are in reference to the NTO scenario.

^a^The residual (i.e. within-participant) variance is reported as an estimated multiplicative factor relative to the scenario NTO. For example, the variance of the residuals for scenario ENF is 2.128 × 0.086 = 0.183.

*p < 0.05 (two-tailed). **p < 0.01 (two-tailed).

The estimated distributions of residuals and random effects were close to a normal distribution, with a Kolmogorov–Smirnov distance (i.e. distance with respect to the supremum-norm) of 0.075 and 0.076, respectively. The residuals were slightly positively skewed (i.e. right-tailed, with a skewness of 0.83) and leptokurtic (with a kurtosis of 5.91), and the random effects were unskewed (skewness of −0.04) and slightly platykurtic (slightly light tails, with a kurtosis of 2.65). Heterogeneity in Δ*RT* variance was observed across scenarios (Figure 9). This variability supports the use of a heteroscedastic model structure to appropriately account for non-constant variance across scenarios. The estimated standard deviation of the random effect of participant was 0.073, corresponding to an estimated ICC of 0.294. This indicates that approximately 29.4% of the total variance in Δ*RT* is attributable to between-participant differences (after removing the effect of *Surprise-I*, *Startle-I,* and *Scenario*), supporting the application of mixed-effects model to account for the non-independence of observations.

When controlling for the effect of Startle-I, Surprise-I, and individual differences, ENF, FLAP, and CARGO resulted in significantly higher Δ*RT* than the reference scenario NTO. In ENF, Δ*RT* was 257 ms higher, *β* = 0.257, *SE* = 0.053, *t*(171) = 4.849, *p* < 0.001. In FLAP, Δ*RT* was 131 ms higher, *β* = 0.131, *SE* = 0.060, *t*(171) = 2.181, *p* = 0.031, and in CARGO, Δ*RT* was 137 ms higher, *β* = 0.137, *SE* = 0.058, *t*(171) = 2.379, *p* = 0.019. Participants showed significantly lower Δ*RT* in LTS and NLO compared to the NTO. Δ*RT* was 107 ms lower in LTS, *β* = −0.107, *SE* = 0.046, *t*(171) = −2.315, *p* = 0.022, and Δ*RT* was 45 ms lower in NLO, *β* = −0.045, *SE* = 0.023, *t*(171) = −1.981, *p* = 0.049.

### Relationships Between Startle and Surprise with ΔAC

The sequence of scenarios had a significant effect on Δ*AC*. *Sequence* was therefore included in the linear mixed-effects model of Δ*AC*:

ΔAC=1+Startle−I+Surprise−I+Scenario+Sequence+(1|ID).

Results from the linear mixed-effects model of Δ*AC* are presented in Table 4. The model revealed no significant effect of *Surprise-I* nor *Startle-I* on Δ*AC*.

**Table 4.** Summary of the linear mixed-effects model of the Delta Accuracy

Effect	Estimate	*SE*	*t*	*p*	Variance factor^a^	Effect size
Fixed effects						
(Intercept)	−0.594	2.845	−0.209	0.835	-	−0.063
Startle-I (1–5)	−0.840	1.267	−0.663	0.508	-	−0.089
Surprise-I (1–5)	−0.640	0.861	−0.744	0.458	-	−0.067
Sequence	0.197	0.304	0.646	0.519	-	0.021
Scenario_ENF_	−25.412	6.038	−4.208	<0.001**	5.447	−2.677
Scenario_FLAP_	−8.115	4.123	−1.968	0.051	3.149	−0.855
Scenario_CARGO_	−8.629	4.734	−1.823	0.070	3.994	−0.909
Scenario_LTS_	−0.745	3.235	−0.230	0.818	1.324	−0.079
Scenario_PFDF_	1.707	2.627	0.650	0.517	1.494	0.180
Scenario_STALL_	−4.710	4.114	−1.145	0.254	2.937	−0.496
Scenario_NLO_	0.053	2.623	0.020	0.984	2.437	0.006
Random effect (*SD*)						
Between-participant	4.498	-	-	-	-	-
Within-participant (NTO)	5.506	-	-	-	-	-

*Note.* Number of observations = 208; coefficients for each Scenario are in reference to the NTO scenario.

^a^Estimated multiplicative factor of the residual (i.e. within-participant) variance with respect to the reference scenario NTO. For example, the variance of the residuals for scenario ENF is 5506 × 5.447 = 29.991.

*p < 0.05 (two-tailed); **p < 0.01 (two-tailed).

The estimated distributions of residuals and random effects were close to a normal distribution, with a Kolmogorov–Smirnov distance (i.e. distance with respect to the supremum-norm) of 0.162 and 0.196, respectively. Both residuals and random effects were negatively skewed (i.e. left-tailed, with skewness −1.56 and −1.75, respectively) and leptokurtic (slightly fat tails, with kurtosis of 8.63 and 6.76). Heterogeneity in Δ*AC* variance across scenarios (Figure 10) further supports modelling heteroscedasticity to account for variance differences between scenarios. The estimated standard deviation of the random intercept was 4.498, corresponding to an approximately ICC of 0.225. This finding shows that approximately 22.5% of the total variance in Δ*AC* stems from between-participant differences, further justifying the use of a mixed-effects model.

The fixed-effects intercept was not statistically significant, indicating that Δ*AC* in NTO was not significantly different from zero. Using NTO as reference, ENF exhibited significantly lower Δ*AC*. Δ*AC* in ENF was −0.594 - 25.412 = −26.006, with *β* = −25.412, *SE* = 6.038, *t*(172) = −4.208, *p* < 0.001.

### Descriptive Statistics

One case of Δ*RT* was missing in ENF and one case in CARGO, due to insufficient numbers of correct responses.

**Figure 7.**
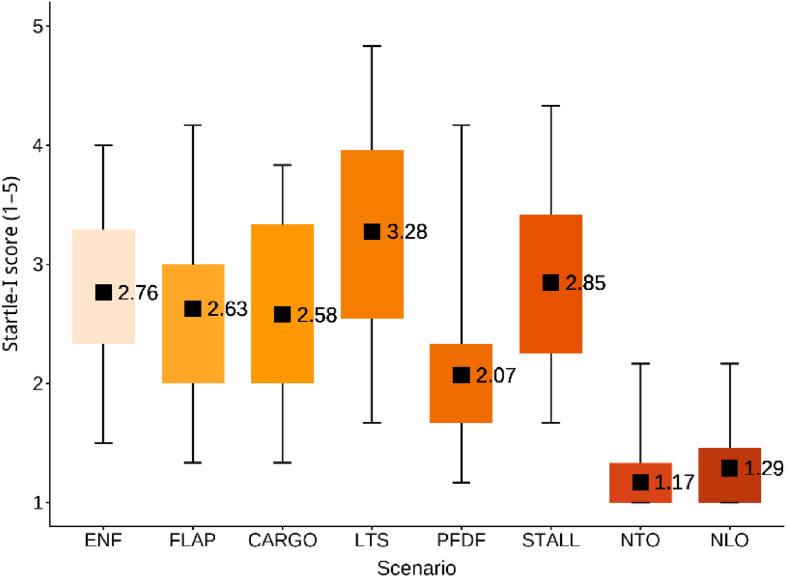
The Startle-I scores in the eight scenarios (square markers indicate means, whiskers indicate interquartile ranges)

**Figure 8.**
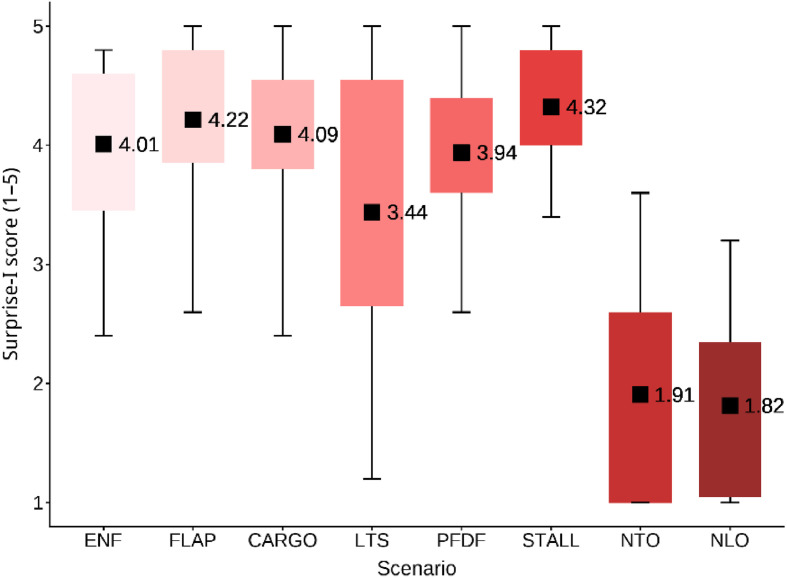
The Surprise-I scores in the eight scenarios (square markers indicate means, whiskers indicate interquartile ranges)

Table 5 lists an overview of dependent measures across the eight scenarios. Figures 7 and 8 present box plots illustrating Startle-I and Surprise-I responses, respectively, across these scenarios. The figures demonstrate that the scenarios elicited high individual variability in startle and surprise responses, although the means of each scenario did not always match our expectations. LTS was not rated very low on surprise, ENF, FLAP, and CARGO resulted in somewhat similar mean startle ratings. In general, Surprise-I scores were higher than Startle-I scores. As expected, LTS produced relatively high startle and low surprise, while STALL produced relatively high startle as well as surprise. PFDF scored as expected relatively low on startle and high on surprise.

**Table 5.** Means and standard deviations of the dependent measures across scenarios

Scenario	Δ*RT* (s)	Δ*AC* (%)	Startle-I (1–5)	Surprise-I (1–5)
	Mean (*SD*)	Mean (*SD*)	Mean (*SD*)	Mean (*SD*)
ENF	0.46 (0.21)	−30.00 (29.26)	2.76 (0.66)	4.01 (0.71)
FLAP	0.33 (0.25)	−12.69 (18.45)	2.63 (0.76)	4.22 (0.65)
CARGO	0.33 (0.25)	−13.08 (22.59)	2.58 (0.75)	4.09 (0.65)
LTS	0.12 (0.13)	−5.38 (8.11)	3.28 (0.93)	3.44 (1.10)
PFDF	0.17 (0.14)	−2.31 (9.08)	2.07 (0.61)	3.94 (0.62)
STALL	0.29 (0.23)	−9.62 (16.37)	2.85 (0.81)	4.32 (0.47)
NTO	0.13 (0.11)	−1.92 (5.67)	1.17 (0.31)	1.91 (0.86)
NLO	0.09 (0.09)	−1.92 (10.96)	1.29 (0.37)	1.82 (0.74)

*Note.* Δ*RT* = Delta Reaction Time; Δ*AC* = Delta Accuracy; Startle-I = Startle Inventory; Surprise-I = Surprise Inventory.

**Figure 9.**
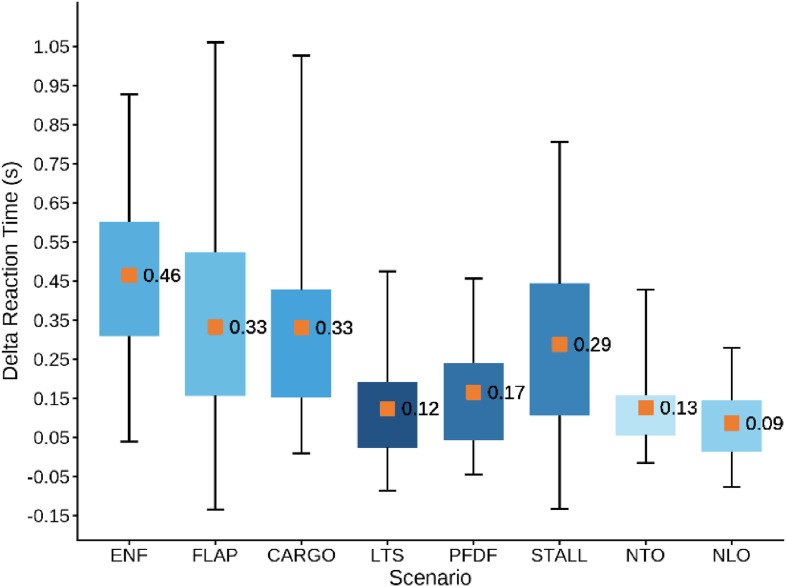
The Delta Reaction Time of the auditory task in the eight scenarios (square markers indicate means, whiskers indicate interquartile ranges)

**Figure 10.**
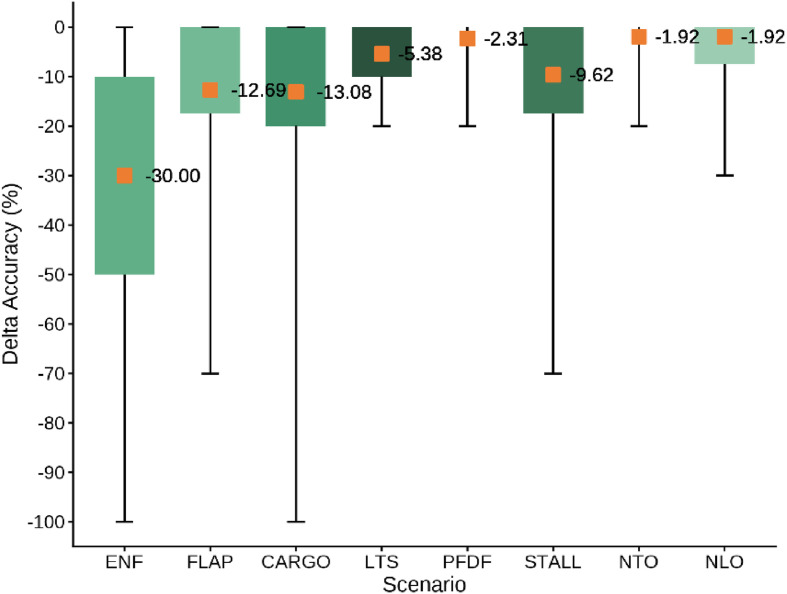
The Delta Accuracy of the auditory task in the eight scenarios (square markers indicate means, whiskers indicate interquartile ranges)

Figures 9 and 10 present box plots of Δ*RT* and Δ*AC* for each scenario. ENF, FLAP, and CARGO, each requiring manual intervention, showed the highest mean Δ*RT*s, lowest mean Δ*AC*s, and the highest variance in both measures. Although STALL did not involve manual intervention, it appeared similarly impactful. In contrast, LTS (highly startling, less surprising) and PFDF (highly surprising, less startling) showed relatively low impact.

### Exploratory Correlation Analyses

Table 6 lists the Pearson correlations between Age, Flight hours, Δ*RT*, Δ*AC*, Startle-I, and Surprise-I scores at between-participant, between-scenario, and residual levels.

**Table 6.** Correlation matrices of the study variables

	Age	FH	Δ*RT*	Δ*AC*	Startle-I	Surprise-I
Between-participant correlation matrix						
Age	1.000					
FH	0.917**	1.000				
Δ*RT*	−0.596**	−0.599**	1.000			
Δ*AC*	−0.107	−0.118	−0.401*	1.000		
Startle-I	−0.242	−0.301	0.237	−0.041	1.000	
Surprise-I	0.097	0.112	−0.038	−0.152	0.134	1.000
Between-scenario correlation matrix						
Age	-					
FH	-	-				
Δ*RT*	-	-	1.000			
Δ*AC*	-	-	−0.932**	1.000		
Startle-I	-	-	0.528	−0.508	1.000	
Surprise-I	-	-	0.718*	−0.543	0.817*	1.000
Residual correlation matrix						
Age	-					
FH	-	-				
Δ*RT*	-	-	1.000			
Δ*AC*	-	-	−0.161*	1.000		
Startle-I	-	-	0.162*	0.008	1.000	
Surprise-I	-	-	0.026	−0.044	0.161*	1.000

*Note.* FH = Flight hours; Δ*RT* = Delta Reaction Time; Δ*AC* = Delta Accuracy; Startle-I = Startle Inventory; Surprise-I = Surprise Inventory.

**p < 0.01 (two-tailed); *p < 0.05 (two-tailed).

At the between-participant level, both Age and Flight hours were significantly negatively associated with Δ*RT*, *r* = −0.596 and *r* = −0.599, respectively. These findings suggest that older and more experienced pilots demonstrate lower Δ*RT* on the secondary task.

At the between-scenario level, Surprise-I was significantly positively correlated with Δ*RT*, *r* = 0.718, and also with Startle-I, *r* = 0.817. These findings suggest that, within the context of the tested scenarios, the relatively more surprising scenarios also tended to be relatively more startling, and were associated with higher Δ*RT* on the secondary task. There was no significant correlation between Startle-I and Δ*RT* on the between-scenario level.

Additionally, Δ*AC* showed a significant negative correlation with Δ*RT* at between-scenario, *r* = −0.932, and between-participant level, *r* = −0.401. These findings indicate that both participants and scenarios with longer Δ*RT* tended to exhibit more errors on the secondary task.

At the residual level, Startle-I was significantly positively correlated with Δ*RT*, *r* = 0.162 and with Surprise-I, *r* = 0.161. This suggests that unusually high Startle-I ratings were associated with higher-than-expected Surprise-I ratings and longer-than expected Δ*RT*. In addition, Δ*AC* showed a significant negative correlation with Δ*RT*, *r* = −0.161. This means that if, in a given scenario, Δ*RT* was higher than expected for that participant and scenario, more errors were likely to occur on the secondary task.

### Temporal Patterns of ΔRT and Valid Responses

**Figure 11.**
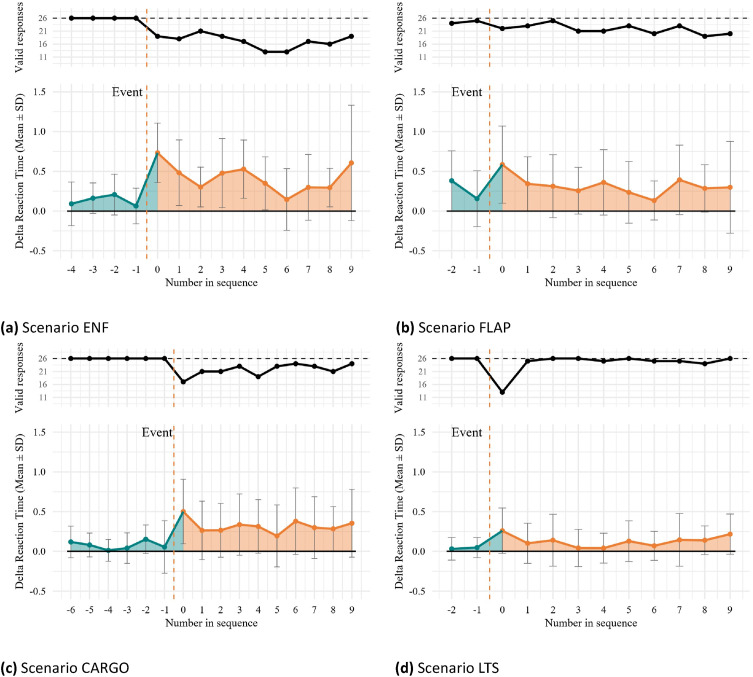
Mean Delta Reaction Time (±1 SD) with valid responses in target block per scenario

**Figure 12.**
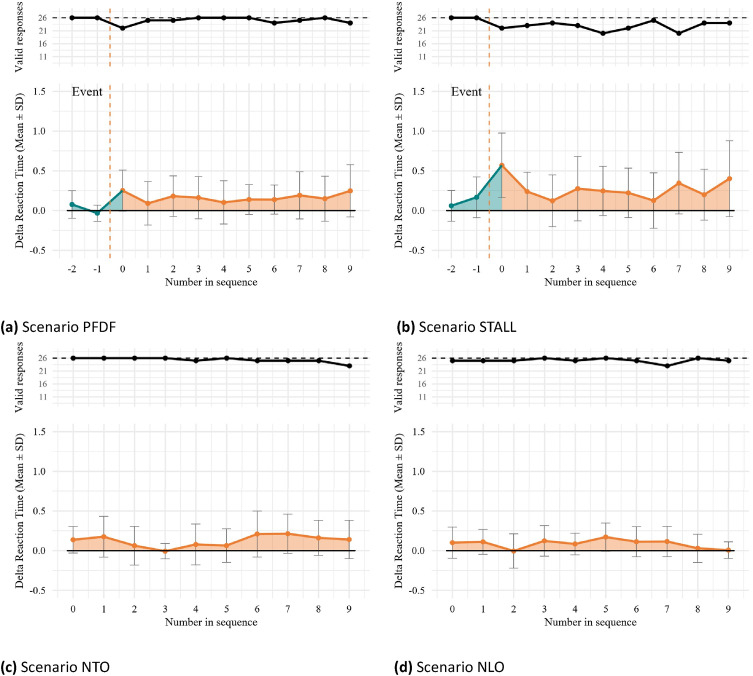
Mean Delta Reaction Time (±1 SD) with valid responses in target block per scenario (continued)

Figures 11 and 12 provide an overview of the Δ*RT* and valid responses across the secondary task duration in the target block. In the Δ*RT* plots, the black line represents a zero change in Reaction Time, while the shaded areas highlight deviations from this reference. In valid responses plots, the gray dashed line indicates the total number of participants (N = 26).

In all scenarios with preset events, Δ*RT* showed a sharp increase immediately after the event (dashed vertical line), peaking at the first number in the sequence and coinciding with a drop in valid responses. For ENF, FLAP, and CARGO, where pilots had to intervene, changes in Δ*RT* and valid responses remained evident throughout the entire measurement period. Additionally, these scenarios exhibited greater postevent variability in Δ*RT*, as reflected by larger standard deviations. A similar pattern was observed in STALL, possibly due to the persistent warning sound. The impact of LTS and PFDF subsided quickly, suggesting a brief impairment in information processing.

## Discussion

We tested whether there were significant relationships between pilots’ startle and surprise responses and information-processing performance during simulated in-flight events, while controlling for general differences between these events, and for general differences between individuals. Our results indicate that more severe startle responses were significantly related to larger impairments of information-processing speed, as evidenced by increased Δ*RT* on the secondary auditory task (see Table 3). This was not additionally reflected in a significant positive correlation between startle and secondary task performance on the scenario level (see Table 6), meaning that other differences between scenarios, such as workload or manual control, overruled this effect when comparing scenarios. The findings align with prior research in lab settings (Duchevet et al., 2025) and in-flight simulators (Martin et al., 2015) suggesting that startle leads to temporary cognitive disruption, likely due to the rapid engagement of survival circuits that prioritize threat responses over executive functioning (Eysenck et al., 2007; LeDoux, 2012). The involuntary nature of startle appears to divert attentional resources from ongoing tasks, requiring additional cognitive demands to reorient focus, which is supported by evidence of increased cerebral blood flow in prefrontal cortex regions (Parasuraman & Caggiano, 2005).

Interestingly, these findings stand in contrast to recent research (Schwartz et al., 2025), which focused on a controlled cognitive task in a single-task paradigm. In this context, no performance impairment following startle was observed, and slight performance improvements were even reported under high cognitive load conditions. One possible explanation for this discrepancy lies in the differences between controlled laboratory environment and the more complex task in our study. In laboratory contexts, the absence of perceived threat may enable participants to rapidly compensate for the effect of startle, limiting the disruptive effect on ongoing tasks. However, real-world startle responses are more likely to occur in highly demanding and threatening situations, where task complexity and pressure may overwhelm compensatory mechanisms and exacerbate performance disruptions.

Contrary to our hypothesis, but in line with a previous study (Duchevet et al., 2025), surprise did not have a distinct significant impact on secondary task performance. While surprise has been theorized to impose cognitive demands by prompting a need for sensemaking and situational “reframing” (Klein et al., 2007; Landman et al., 2017), our results show that its immediate effect on information processing were not significant in the in-flight events featured in our experiment. It could be that the surprising events in our study may not have been complex enough to elicit sufficient variance in pilots’ “reframing” efforts that would induce sufficient additional workload to impact cognitive task performance. This discrepancy highlights the complexity of modelling surprise effects and suggests that its impact may be more contextual, indirect, or delayed, compared to the more reflexive and acute nature of startle.

Although the mixed-effects model did not reveal a significant relationship between surprise and information-processing performance, this does not imply that surprise is less relevant for pilot training. In fact, a significant correlation between surprise ratings and Δ*RT* was observed at the between-scenario level, suggesting that more surprising scenarios are generally associated with greater disruption to information-processing performance. Additionally, the temporal pattern analysis highlights the influence of surprise on response dynamics. One possible explanation for this discrepancy is that the between-scenario correlations reflect general trends across scenarios, whereas the mixed-effects model accounts for both between-participant and between-scenario variability while controlling for other contributing factor. After adjusting for startle ratings, scenario level variability, and individual differences, the effect of surprise on Δ*RT* and Δ*AC* was non-significant. Nevertheless, previous studies have shown that unexpectedness in training is important to build cognitive flexibility and higher-level competences to deal with a wide variety of possible events (Helmreich et al., 2017; Landman et al., 2018; Salas et al., 2006). Given its importance for training, more research is needed into means to systematically introduce surprise in aviation scenarios in a highly ecologically valid manner.

There was a negative correlation between flight experience and Δ*RT*, suggesting that more experienced pilots were better at managing cognitive disruptions caused by in-flight events. This finding supports the notion that expertise and training can mitigate the impact of unexpected events (Causse et al., 2019), potentially by enabling pilots to rely on well-practiced procedural knowledge that requires fewer cognitive resources under stress. This underscores the importance of tailored training programs designed to enhance resilience to startle, particularly for less experienced pilots.

The findings further highlight the necessity of incorporating startle management strategies into pilot training programs. Although current UPRT protocols acknowledge the role of startle and surprise in aviation safety, our research suggests that targeted interventions specifically designed to mitigate startle-induced cognitive disruptions may be beneficial (Vlaskamp et al., 2024). Techniques such as stress management, exposure-based training, and cognitive resilience exercises may help pilots reduce the disruptive effects of startle and maintain optimal performance under pressure (Causse et al., 2011; Driskell & Johnston, 1998; Landman et al., 2020).

Several limitations of the current study should be acknowledged, along with corresponding recommendations for future research. First, although the data revealed both within-scenario and between-scenario variation in Startle-I ratings, the intensity of the startle responses observed was likely lower than those typically experienced during real-world aviation emergencies. This limitation may be attributed to the controlled nature of the simulation context, in which participants were aware that no actual threat was present. Future research could employ more immersive simulation techniques, introduce social stressors (e.g. performance evaluation and peer observation) or incorporate sudden-onset stimuli to elicit stronger startle responses, and further validate their effects under more ecologically intense conditions.

Second, the quantification of startle and surprise relied on self-report measures, which, although informative and psychometrically validated, are subject to potential biases such as individual differences in interpreting questionnaire items. Future research could enhance the assessment of startle and surprise by integrating self-report data with physiological indicators (e.g. reflex electromyogram (EMG) (Blumenthal et al., 2005), pupillometry (Leuchs et al., 2019), and heart rate (Kinney and O’Hare, 2020)).

Third, the experimental setup involved single-pilot crew operating a twin-propeller model, which may not fully replicate the dynamics of real-world multi-crew operations. Investigating how startle and surprise interact in team-based settings could provide valuable insights into crew resource management strategies and training.

Fourth, certain scenario characteristics, most notably the requirement for manual control, were inherently confounded with scenario membership. As a consequence, manual control could not be modelled as an independent predictor alongside scenario effects without introducing perfect collinearity. Nevertheless, startle was assessed at the individual level and exhibited substantial variability within and across scenarios, allowing its association with Delta Reaction Time to be estimated beyond scenario-level differences. Overall, these results indicate that secondary task performance decrements likely reflect the combined influence of startle responses and task-related demands (e.g. manual control). Future studies could address this limitation by directly measuring control activity or by experimentally manipulating manual control independently of scenario context.

Fifth, the lack of significant findings regarding Δ*AC* might be due to low resolution of the secondary task measurement or ceiling effects. Future studies might benefit from integrating additional metrics, such as physiological measures or increasing the difficulty of the cognitive task, to better capture the fluctuation of information-processing performance.

## Conclusion

In summary, the study demonstrates that higher levels of startle were significantly associated with impaired information-processing performance in pilots, whereas no such association was found for surprise. These findings reinforce the importance of training interventions aimed at managing startle effects in high-risk environments. Additionally, the role of experience in mitigating these effects highlights the value of continued practice and exposure-based training. Future research should explore more nuanced measures of cognitive capacity and extend these findings to multi-crew settings to further enhance aviation safety protocols.

## Key Points

• Higher levels of startle, as measured by self-report, was significantly associated with impaired information-processing speed, whereas no such association was found for surprise. The information-processing performance were assessed using a secondary auditory cognitive task.
• The study employed eight different in-flight scenarios in a highly ecologically valid setting.
• The flight scenarios induced an adequate range of startle and surprise responses in the pilots.
• The findings highlight the need for tailored interventions and training protocols to mitigate the potentially disruptive effects of startle responses, enhancing pilot performance during in-flight emergencies.
